# Influence of Cherry Cultivar and Ethanol Concentration on the Oenological Properties of Fermented Cherry Wines

**DOI:** 10.3390/molecules31091382

**Published:** 2026-04-22

**Authors:** Cong Wang, Miaomiao Li, Liang Li, Xutao Wang, Bo Li, Yang Yu

**Affiliations:** 1 College of Food Science and Engineering, Shandong Agriculture and Engineering University, Jinan 250100, China; wangcblb@163.com (C.W.);; 2 Zibo Tasman Brewing Raw Materials Co., Ltd., Zibo 255000, China

**Keywords:** cherry cultivars, alcohol content, physicochemical properties, antioxidant activity, volatile compounds

## Abstract

Four sweet cherry cultivars (FuChen, Redlight, Huangmi, and Samituo) grown in northern China were used to produce sweet cherry wines with two alcohol levels. Physicochemical properties, antioxidant capacity, and volatile aroma compounds of the wines were systematically investigated. The results showed that wine from the Redlight cultivar with an alcohol content of 11.22 ± 0.17% contained the highest phenolic content and also exhibited the strongest antioxidant capacity as measured by DPPH and ABTS•^+^ assays. Meanwhile, wine from the FuChen cultivar with an alcohol content of 11.45 ± 0.03% had the highest anthocyanin content and showed the strongest FRAP antioxidant activity. Orthogonal partial least squares discriminant analysis (OPLS-DA) based on electronic nose data clearly distinguished the eight sweet cherry wine samples from different cultivars. A total of 58 volatile compounds were identified by headspace solid-phase microextraction coupled with gas chromatography–mass spectrometry (HS-SPME-GC-MS). Both principal component analysis (PCA) and OPLS-DA revealed clear differences among the sweet cherry wines based on their volatile composition. Using variable importance in projection (VIP) scores > 1 and relative odor activity values (ROAVs), the key aroma compounds contributing to the characteristic aroma profiles of the eight sweet cherry wines were identified as ethyl butanoate, isoamyl acetate, isoamyl hexanoate, methyl decanoate, ethyl decanoate, ethyl benzoate, methyl salicylate, citronellol, and eugenol. These findings provide important guidance for the selection of raw materials to improve the production of sweet cherry wines with targeted alcohol levels.

## 1. Introduction

The sweet cherry (*Prunus avium* L.) is widely cherished by consumers for its distinctive taste and rich nutritional profile. It contains an abundance of micro- and macro-nutrients, such as carbohydrates, amino acids, vitamins, organic acids, minerals, and various bioactive components like flavonoids, phenolic acids, and anthocyanins [1,2]. Several fruits, such as strawberries and core fruits (apricots, plums, and cherries), were tested for their potential as substrates for producing novel fruit wines rich in phenolic compounds, and the results showed that sweet cherries (Burlat cultivar) were the favorite due to their high phenolic content and effective anti-DPPH free-radical activity [3]. The Burlat cultivar displayed novel medicinal properties and exerted good health benefits [4,5]. However, cherries have a limited harvesting period, a short shelf life, and are prone to damage during transportation, which limits the development of the cherry industry. Therefore, the need for longer processing of cherries, which extends its industrial chain, has become increasingly a point of concern. At present, European countries have a well-developed cherry industry, and products for this industry comprise mature cherry jam, fruit juice, fruit wine, and brandy products.

Cherry fruit has been used to make wine for many years. Today, cherry wine is becoming more popular and increasingly consumed in China, especially in the northeast region [6]. Most existing research has primarily concentrated on fermentation conditions and aroma analysis through various techniques [7,8]. In contrast, studies comparing the quality of wines made from different cherry cultivars are rare, especially low-alcohol cherry wines. Fruit wines with low alcohol content, specifically 1–7% (*v*/*v*), are garnering interest due to their social and health implications [9]. Low alcohol may produce unbalanced wine products with high acidity, unfermented juice, and bad-aroma fermented compounds [10]. It has also been reported that high-alcohol-content wines are generally unbalanced with a bad aroma and low fruitiness, and the main aroma is usually masked [11]. The characteristics of fruit wines differ notably across various cultivars. Physical and chemical attributes, such as hydrogen ion concentration, total acidity that can be titrated, hue, the amount of soluble solids, biologically active substances, and volatile components, are crucial elements in deciding the quality of cherry wine [12,13]. Therefore, research on the disparity in alcohol content and the quality of wine among different cherry fruit cultivars is central to the production of high-quality cherry wine.

Yantai, located in Shandong Province, is known as the “Town of Big Cherry in China”. It is the main sweet-cherry-producing area in China, with the earliest cultivation, the largest industrial scale, and the highest yield. In this study, four cherry cultivars, namely FuChen, Redlight, Huangmi, and Samituo, were selected. The aims of this research were to assess the impacts of varying alcohol contents and cherry cultivars on the quality and aroma of cherry wine and to determine appropriate cherry cultivars. The physicochemical features, nutritional content, and antioxidant capacity of different cherry wines were investigated. The volatile compound profiles of eight cherry wines derived from four cultivars were investigated by means of an electronic nose and a technique integrating headspace solid-phase micro-extraction (HS-SPME) with gas-chromatographic–mass-spectrometric (GC-MS) analysis. A comprehensive multi-index assessment approach was employed. It encompassed hierarchical clustering, principal component analysis (PCA), orthogonal partial least squares discriminant analysis (OPLS-DA), and relative odor activity value (ROAV). This approach facilitated the analysis of differences in volatile compounds and the identification of the crucial active substances that endow cherry wines with their unique aroma.

## 2. Results and Discussion

### 2.1. Physicochemical Properties of the Various Cherry Wines

The physicochemical properties of the various cherry wines prepared are shown in Table 1. The FC-6, RL-6, HM-6, and SMT-6 wines, with alcohol contents ranging from 6.0% to 7.0% (*v*/*v*), were classified as low-alcohol wines. All cherry wine samples, categorized as dry fruit wines, exhibited reduced sugar contents below 4 g/L. High-alcohol wines generally showed slightly higher total acid content compared to their low-alcohol counterparts within the same cultivar. Nonetheless, there was no notable variance in the overall acidity levels between SMT-6 and SMT-11 wines. Cherry wines exhibited higher total acid content compared to grape wines. Acidity plays a crucial role in wine quality, contributing to its freshness and overall sensory profile [14]. However, many people do not prefer very-high-total-acid-content wine. Compared with other wine samples, the SMT wines (SMT-6 and SMT-11) had the lowest pH (3.56 ± 0.02, 3.60 ± 0.02) and highest total acid content (8.48 ± 0.05 g/L, 8.47 ± 0.05 g/L). The FC-6 and FC-11 wines had lowest total acid content of 7.11 ± 0.06 g/L and 7.99 ± 0.06 g/L, respectively. The results of our study offer a theoretical foundation for proper regulation of total acid content in wine and the basis for selecting the best cherry cultivars for wine preparation. Although different cherry cultivars were used, the final pH values were maintained between 3.56 ± 0.02 and 3.85 ± 0.01. The pH varies across different cherry cultivars but is mainly between 3.60 and 4.50 [15]. The volatile acid content of different cherry wines ranged from 0.39 ± 0.02 to 0.45 ± 0.02 g/L. They contribute to the flavor by interacting with other flavonoid compounds in wine within the concentration range of 0.20–0.70 g/L [16].

Statistical analysis revealed significant disparities in total phenolic content (TPC) and total flavonoid content (TFC) across the eight wine specimens (*p* < 0.05), as depicted in Table 1. The red cherry wines (FC and RL) had significantly higher amounts of total phenolic content (TPC), total flavonoid content (TFC), and total anthocyanin content (TAC) than the yellow cherry wines (HM and SMT). The TPC in the wines varied from 628.23 ± 5.69 to 900.90 ± 3.41 mg GAE/L, the TFC ranged from 40.00 ± 1.11 to 82.20 ± 0.36 mg CAE/L, and the TAC was between 5.11 ± 0.47 and 21.25 ± 1.25 mg/L. In the study by Xiao et al. [7], the TPC in nine cherry wines ranged from 235.53 to 736.54 mg GAE/L. The TAC in cherry juice of the Tieton cultivar was 31.3 mg/L but decreased to 5.1 mg/L after fermentation [17]. The TPC, TFC, and TAC contents were higher in high-alcohol wines than in low-alcohol wines fermented from the same cultivar. The highest values were found in RL-11 wine, with TPC at 900.90 ± 3.41 mg GAE/L and TFC at 82.20 ± 0.36 mg CAE/L, and in FC-11 wine, with TAC at 21.25 ± 1.25 mg/L. The lowest values were observed in SMT-6 wine, with TPC at 628.23 ± 5.69 mg GAE/L and TAC at 5.11 ± 0.47 mg/L, and in HM-6 wine, with TFC at 40.00 ± 1.11 mg CAE/L. The results showed that the alcohol content in wine significantly influences TPC, TFC and TAC (*p* < 0.05). The vinification procedure had effect on TPC, with the highest TPC observed in the sweet cherry wines prepared with sugar and pits [3]. Throughout the winemaking process, the phenolic compound profiles in fruits undergo transformations and partial degradation during fermentation [18]. Phenolic compound contents varied significantly among cherry wines produced from different cultivars. Anthocyanins, the predominant phenolic compounds in cherries and a key contributor to wine quality, were detected in all eight cherry wine samples [18,19]. However, a poor correlation exists between TAC and TPC, because anthocyanins react poorly with Folin–Ciocalteu reagent [20].

### 2.2. Antioxidant Capacity and Correlation Analysis

**Figure 1:**
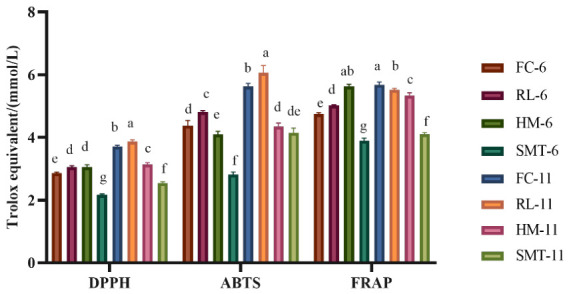
The antioxidant activity of eight cherry wines. Values are presented as mean ± standard deviation (SD) of three replicas. Different letters (a–g) indicate significant differences among groups (*p* < 0.05) based on the Duncan test.

There are many ways to determine antioxidant capacity, and several methods are often preferred because a single method is rarely sufficient, particularly for complex substances. In this study, we incorporated three methods (DPPH, ABTS^+^•, and FRAP) into our analysis, and the outcomes are presented in Figure 1. All three methods revealed significant differences in the antioxidant activity among wines prepared from the same cherry cultivar, and similar trends of the three methods were observed in these eight wines. Under the DPPH and ABTS^+^• analyses, RL-11 showed the highest antioxidant activity, with values of 3.86 ± 0.06 mmol Trolox/L and 6.06 ± 0.23 mmol Trolox/L, respectively, while SMT- 6 had the lowest, at 2.17 ± 0.03 mmol Trolox/L and 2.81 ± 0.09 mmol Trolox/L. FRAP, for its part, showed that the antioxidant activity was highest (5.67 ± 0.09 mmol Trolox/L) in FC-11 wine and lowest in SMT-6 wine (3.89 ± 0.08 mmol Trolox/L). As shown in Figure 1, the antioxidant capacity of wines fermented from red cherries was higher than that of the yellow cultivar. For the same cherry cultivar, it was higher in high-alcohol than in low-alcohol cherry wine.

As shown in Table 2, TPC strongly correlated with the ABTS^+^• scavenging ability of the wines (*p* < 0.01). However, the FRAP assay indicated a non-substantial relationship among the total phenolic content (TPC) and the wine’s antioxidant capacity. ABTS^+^ revealed that TFC contributed to the scavenging ability of cherry wines, whereas DPPH revealed that TFC had no significant effect on the scavenging ability of cherry wines. Similarly, FRAP revealed that TFC had no significant effect on the antioxidant ability of cherry wines. ABTS^+^• and DPPH showed that TAC strongly correlated with the scavenging ability of cherry wines (*p* < 0.01). In contrast, FRAP showed that no correlation existed between TAC and the scavenging ability of cherry wines. A previous study using DPPH and ABTS methods revealed that TPC and TFC in yellow peach wine correlated with antioxidant activity. In contrast, FRAP revealed no significant difference in the antioxidant activity among yellow peach wines with different TPC and TFC levels [21]. Certain research studies have indicated a strong correlation between the total phenolic content (TPC) [22,23] and antioxidant activity in fruit wines. However, other research has found no notable correlation between them [3,24,25].

### 2.3. Electronic Nose Data

**Figure 2:**
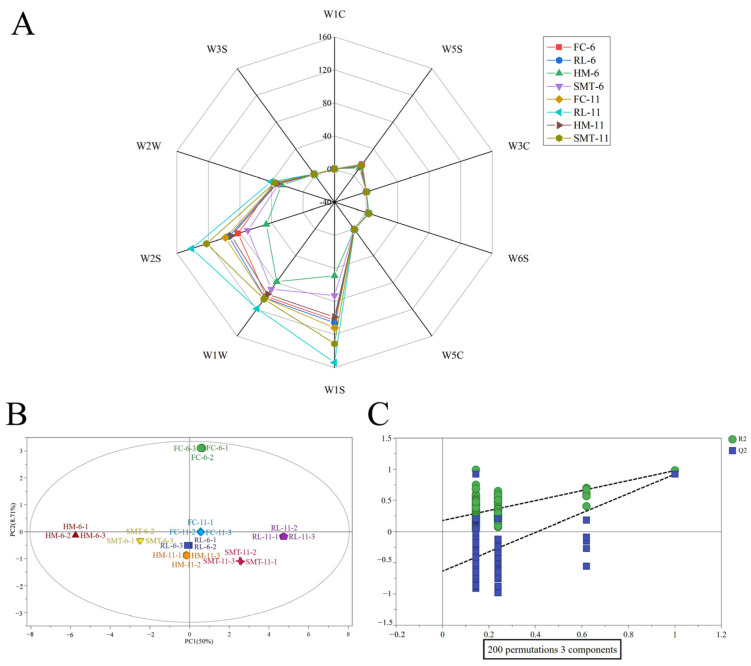
Radar fingerprint (**A**), OPLS-DA score plot (**B**), and permutation test (**C**) based on electronic nose responses of eight sweet cherry wines (*Prunus avium* L.).

An electronic nose was employed to examine the existence and quantity of volatile compounds in the eight cherry wine samples. Combined with the data in Supplementary Table S2, the electronic nose characteristic odor radar fingerprinting map is drawn (Figure 2A). Several differences in the response existed among the samples, but the map contour was the same across them. The main volatile components (alcohols, aromatic) were higher in W2S and W1S, while terpenoids and sulfides were present in W1W.

Based on the response values of different electronic nose sensors, OPLS-DA was further performed (Figure 2B). Eight cherry wine samples could be effectively distinguished. With R^2^X (0.805), R^2^Y (0.572), and Q^2^ (0.823) all surpassing the 0.5 acceptability criterion [26], the model exhibited robust predictive capability. Figure 2C further validates model reliability through permutation testing, showing the Q^2^ regression intercept below zero after 200 iterations aligning with permutation test protocols described by Good [27]. There was also no overlap between the different wine samples, indicating that certain differences existed in the aroma of cherry wines of different cultivars and alcohol contents. Among the 10 sensors, four sensors with VIP > 1 (W5S, W6S, W5C and W2W) were considered to contribute more to the OPLS-DA model in this experiment. Subsequently, gas chromatography–mass spectrometry (GC-MS) was utilized to identify and measure the different volatile elements, based on the electronic nose evaluation of differences within the overall fragrance characteristics of cherry wines.

### 2.4. Volatile Compounds Analysis

Supplementary Table S3 lists the types and quantities of volatiles in various cherry wine samples. It identifies 58 main components, such as 14 alcohols, 29 esters, 5 acids, 5 terpenes and lactones, 2 aldehydes, 2 phenols, and 1 ether. A total of 51, 53 51, 52, 53, 54, 49, and 53 volatile compounds were identified in FC-6, RL-6, HM-6, SMT-6, FC-11, RL-11, HM-11 and SMT-11, respectively. Significant differences existed in the contents of the aroma-related compounds between different alcohol wines for the same cherry cultivars, and except for the FC cherry cultivar, the overall level of volatiles in low-alcohol wines was less than in high-alcohol samples. This finding suggests that the alcohol content significantly influences the content of volatile components in several cherry wines, and perhaps the FC cherry cultivar is more suitable for producing low-alcohol wine. The highest total contents of volatile compounds were observed in RL-11 (192.93 ± 4.19 mg/L), and the lowest in HM-6 (84.89 ± 2.53 mg/L). As illustrated in Supplementary Figure S1A, the primary volatile constituents in the eight cherry wines were alcohols and esters, aligning with the results reported by Niu [6] and Li [28].

Esters with floral and fruity aromas are mainly derived from fruit and ethanol fermentation and greatly influence wine aroma [29]. A total of 29 esters, comprising acetate, ethyl esters of short-chain fatty acids, and ethyl esters of medium-chain fatty acids, were found in eight wine samples (Supplementary Figure S1B). Among them, 27, 26, 25 and 29 ester compounds were detected in FC-6, RL-6, HM-6 and SMT-6, respectively, while 29, 28, 28 and 27 esters were detected in FC-11, RL-11, HM-11 and SMT-11, respectively. Ester contents were highest in FC-6 (108.13 ± 4.17 mg/L) and lowest in HM-6 35.97 ± 2.05 mg/L). With the exception of the FC cultivar, the total ester contents were lower in the low-alcohol than in the high-alcohol wine fermented from the same cherry cultivar. Acetate has strawberry, banana, pear and other fruit flavors and a sweet floral smell. The highest acetate concentration was found in SMT-11 (21.27 ± 1.29 mg/L), and the isoamyl acetate content had the highest value (19.77 ± 1.54 mg/L). The ethyl ester of short-chain fatty acids detected was mainly ethyl butanoate, which has a light fruity flavor (strawberry). Its concentration in SMT-11 (1.58 ± 0.32 mg/L) was significantly higher than in other groups, and it was only detected in HM and SMT, two yellow cultivars of cherry. Ethyl acetate and ethyl butanoate are key compounds found in strawberry and strawberry-flavored grape berries [30,31]. Among the medium-chain esters detected, ethyl hexanoate, ethyl enanthate, ethyl octanoate and ethyl decanoate were particularly prevalent, contributing a fruity and rose aroma to cherry wine. RL-11 wine had the highest concentration of these esters (88.77 ± 3.69 mg/L), with ethyl octanoate and ethyl decanoate standing out. Notably, ethyl octanoate and ethyl decanoate were also prominent in cherry spirits fermented from five Serbian cherry cultivars [32]. Generally, these cherry wines are rich in diverse esters derived from fatty acid ethyl esters, which serve as secondary yeast metabolites to enrich the wine’s fruity character [33].

A total of 14 alcohols were detected in the eight cherry wines. The alcohols were most abundant in RL-11 (79.71 ± 1.12 mg/L) and lowest in SMT-6 (43.910 ± 1.89 mg/L). With the exception of the FC cultivar, the total alcohol contents in low-alcohol wine were lower than in the high-alcohol wines prepared from the same cherry cultivar. Among the alcohols detected, isoamyl alcohol, hexanol, benzyl alcohol, and phenylethyl alcohol were present in higher quantities. Additionally, two C6 compounds, 1-hexanol and (Z)-3-hexenol were also found in cherry wines. Both compounds and terpenes belong to the aroma family, and their components are mainly derived from the hydrolysis of aroma glycoside precursors in the cherry peel, and they play a critical role in giving fruit wine from given regions their characteristic taste [34]. The content of 1-hexanol was higher in HM-11 and HM-6 wines than the rest of the wines. This might explain why the HM cherry cultivar has a strong, unique aroma. The main phenylethyl substance in cherry wine is phenylethanol, which is a derivative of shikimic acid with a characteristic rose flower fragrance [35]. The highest value was found in the SMT-11 wine sample (4.29 ± 0.29 mg/L).

Fatty acids can produce creamy and cheesy flavors at low concentrations but produce sour and rancid taste odors at high concentrations [36]. Five acids were detected in the tested cherry wines, and the most abundant one was octanoic acid, followed by caproic acid, acetic acid, decanoic acid and propionic acid. Octanoic acid is often oily and musty, but at low concentrations, it has a pleasant cheese and fruit aroma [37]. The acids were most abundant in RL-6 and FC-6, at 3.53 ± 0.53 mg/L and 3.22 ± 0.22 mg/L, respectively.

In fruits, terpene compounds generally exist in the form of glycosides. During the brewing process of fruit wine, they form free volatile substances under acid or enzymatic hydrolysis, giving fruit wine a fruity and floral aroma. Thus, they give fruit wines their characteristic aroma. Furthermore, terpene compounds have a strong aroma impact even at low levels, significantly contributing to the scent of fruit wine [38]. Within the group of eight cherry wines, five fragrant compounds were identified, including linalool, citronellol, α-terpineol, geraniol, and butyrolactone. Compared with other cultivars, RL-6 and RL-11 wines had the highest terpene content in low- and high-alcohol wines of the same cherry cultivar, but the terpene content increased with alcohol content. Geraniol has a mint aroma and tropical fruit sweetness, and it was only detected in RL and SMT wines. Benzaldehyde, mainly present in cherry fruits and detected in various cherry cultivars [39], is one of the most important aroma-related compounds for cherry fruit, imparting almond and caramel aromas to wine [29].

### 2.5. Multivariate Statistical Analysis

**Figure 3:**
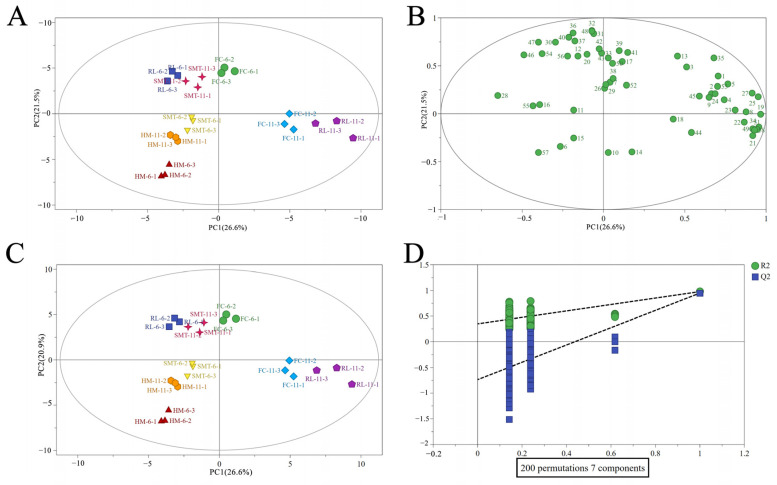
Principal component analysis (PCA) (**A**,**B**) and OPLS-DA (**C**,**D**) of volatile compounds in eight sweet cherry wines (*Prunus avium* L.) analyzed by HS-SPME-GC-MS.

Principal component analysis (PCA) was used to examine the distribution of volatile compounds across various cherry wine samples (Figure 3A,B). The result showed that eight cherry wine samples could be classified into eight distinct groups, suggesting that the wines had a relatively distinct aroma profile. Both cultivar and alcohol content have significant effects on volatile compounds. FC-6, RL-6 and SMT-6 were in distinct groups from HM-6, and FC-11, HM-11, and SMT-11 grouped in a separate cluster from RL-11. HM-6 and RL-11 groups were clustered far apart from other samples, suggesting that a significant difference in the profile of volatile compounds existed between HM-6 and RL-11, and other samples.

The OPLS-DA analysis of the 58 volatile compounds mentioned in Figure 3C revealed performance metrics for the model: Seven-fold cross-validation yielded R^2^X = 0.909, R^2^Y = 0.985, and Q^2^Y = 0.936, all exceeding 0.5, confirming good fitness and predictability of the OPLS-DA model. Additionally, the 200 permutation test (depicted in Figure 3D) demonstrated that both the R^2^ and Q^2^ intercepts fell below their initial points. Remarkably, the intercept of the Q^2^ regression line along the vertical axis remained below 0, which suggests the absence of overfitting in the model. These observations affirm the model’s credibility, ensuring that the results accurately reflect the distinct aroma profiles of different cherry wines. Both the volatile composition analysis and electronic nose response effectively discriminated among the different wine samples, with slight differences observed between the two models.

### 2.6. Comparison of Key Volatile Compounds

**Figure 4:**
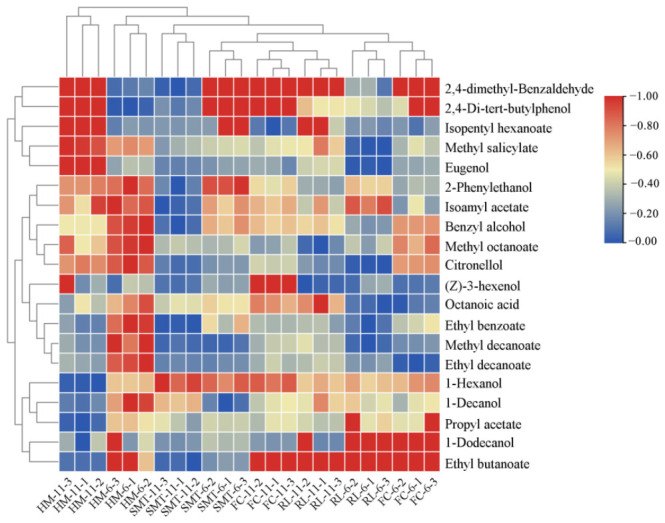
Heatmap of the different volatile compounds in eight cherry wines.

As the VIP value of volatile substances increases, so does their contribution to the general scent. For pinpointing crucial compounds active in scents, a two-pronged method was utilized, focusing on the fluctuating significance in projection (VIP) scores above 1, as deduced from OPLS-DA modeling, and significant differences (*p* < 0.05) confirmed by Kruskal–Wallis analysis. This strategy enabled the selection of 20 volatiles critically associated with the sensory stratification of cherry wine samples. Among them, six alcohols, nine esters, one acid, one terpene, one aldehyde and two phenols were identified. A cluster heatmap was plotted to assess the differences among the 20 volatiles of the eight cherry wines, and four main clusters were obtained (Figure 4).

Volatile compounds that were grouped in cluster 1 were most abundant in HM-11 and were least abundant in SMT-11. The characteristic volatile compounds in cluster 1 were 2,4-dimethyl-benzaldehyde, 2,4-Di-tert-butylphenol, isopentyl hexanoate, methyl salicylate and eugenol, which gave the cherry wine fruity, peppermint, and clove aromas. The volatile compounds in clusters 2 and 3 were most abundant in HM-6 and least abundant in SMT-11. The volatile compounds of clusters 2 and 3, including 2-phenylethanol, isoamyl acetate, benzyl alcohol, methyl decanoate, citronellol, (Z)-3-hexenol, octanoic acid, ethyl benzoate, methyl decanoate and ethyl decanoate, gave the cherry wine fruity and floral aromas. Volatile compounds in cluster 4, including 1-hexenol, 1-Decanol, propyl acetate, 1-Dodecanol and ethyl butanoate, were most abundant in FC-6, RL-6, RL-11 and FC-11, which are red cultivars cherry wines. These volatiles impart fruity and varietal aromas. Different cherry cultivars had different amounts and types of volatile compounds, and the abundance of some of the volatile substances did not increase with alcohol content in low- and high-alcohol wines of the same cherry cultivar.

The ROAV of 20 key compounds that strongly impact the aroma (VIP > 1) was calculated (Table 3). The ROAVmax of isoamyl acetate in SMT-11 cherry wine was assigned a value of 100 as per de Ovalle et al. [40], which imparts a banana-like fruity and floral scent to the wine. Nine compounds, ethyl butanoate, isoamyl acetate, isopentyl hexanoate, methyl decanoate, ethyl decanoate, ethyl benzoate, methyl salicylate, citronellol, and eugenol had ROAV ≥ 1, implying that they were the key and common characteristic volatile compounds in cherry wines. The distribution of the above nine key aroma active compounds varied across the cherry wine cultivars. Compared with other samples, isoamyl acetate (100), ethyl butanoate (12.01), ethyl benzoate (4.71) and eugenol (3.98) in SMT-11 and isopentyl hexanoate (7.91), methyl decanoate (7.78), methyl salicylate (2.76), and citronellol (1.51) in RL-6 were more prominent in ROAV, and most of them impart flower and fruit characteristics. The OAV study emphasized the critical role of eight primary scents—ethyl acetate, ethyl butanoate, ethyl isovalerate, isoamyl acetate, ethyl hexanoate, ethyl octanoate, ethyl decanoate, and ethyl 3-phenylpropanoate. These constituents are recognized as essential aromatic elements in such wines, according to Mara et al. [41]. The variations in the concentrations of these key aroma components are primarily attributed to differences in cultivars and alcohol content. Consequently, optimizing the alcohol concentration can enhance the characteristic aroma of cherry wine.

## 3. Materials and Methods

### 3.1. Materials and Reagents

Several chemicals were bought from Sigma-Aldrich (Shanghai, China). These included DPPH (a free-radical compound), ABTS (a radical cation), TPTZ (a chemical for assays), Trolox (an antioxidant-like compound), Folin–Ciocalteu reagent (used for analysis), 4-methylpentan-2-ol (an alcohol), and a set of n-alkanes from C7 to C30.

Saccharomyces cerevisiae yeast (LALVIN EC-1118) and pectinase (Lafazym Extract, 500 g) were acquired from Lallemand Inc. (Montreal, QC, Canada). Food-grade potassium metabisulfite (K_2_S_2_O_5_) was purchased from China Baiweixiang Biotechnology Co., Ltd. (Beijing, China). All analytical-grade reagents, including sodium hydroxide, Folin–Ciocalteu reagent, ferric chloride, absolute ethanol, sodium acetate and concentrated hydrochloric acid, were purchased from China Sinopharm Chemical Reagent Co., Ltd. (Shanghai, China).

### 3.2. Preparation of Cherry Wines

The four sweet cherry cultivars used in this study, FuChen (FC), Redlight (RL), Huangmi (HM) and Shamituo (SMT), belong to *Prunus avium* L. They were harvested in May 2022 from Yantai City, Shandong Province, China, the main sweet-cherry-producing region in China [47]. Notably, few studies have been reported regarding winemaking using the FuChen cultivar. FuChen is a new sweet cherry cultivar, bred by Yantai Academy of Agricultural Sciences and mainly planted in Yantai. It features early maturity, crisp and firm flesh, as well as moderate sugar and acid contents. The cultivar name, total soluble solids (TSS, °Brix), total sugar, total acidity, and pH values of the fresh cherry samples are listed in Table S1.

Cherry fruits were manually destemmed and pitted. The cherry fruits were crushed and transferred into 5 L fermentation tanks, filling up to two-thirds of the total volume. High-grade sucrose was added in one batch to adjust the sugar content to achieve the target alcohol level. Subsequently, 50 mg/L SO_2_ (supplied by potassium metabisulfite), 0.3 g/L pectinase, and 0.2 g/L active dry yeast were added sequentially.

The cherries were fermented in different ways as follows: Two fermentation processes were employed. In Process 1, cherry pulp was mixed with yeast EC1118, and high-grade sucrose was added. The mixture was fermented to produce dry wines (FC-11, RL-11, HM-11, and SMT-11) with an alcohol content of 11% *v*/*v*. In Process 2, cherry pulp was combined with yeast EC1118 without adding high-grade sucrose (FC-6, RL-6, HM-6, and SMT-6). Low-alcohol cherry wines (FC-6, RL-6, HM-6, and SMT-6) were produced by spontaneous fermentation until the residual sugar content was below 4 g/L. Fermentation was carried out at 25 °C with no commercial yeast nutrients added, and no aeration, acidity or water adjustment, or maceration was applied. All batches fermented normally with no stuck fermentation or H_2_S off-odors detected. After fermentation, the samples were filtered, supplemented with 0.03 g/L SO_2_ (from potassium metabisulfite) for stabilization, naturally clarified for 7 days, and then stored at 4 °C for 1 month. The supernatant was then collected and stored sealed in the dark at −20 °C until instrumental analysis. Each cultivar was fermented in triplicate, with independent fermentation for each replicate.

### 3.3. Physicochemical Analysis

The TSS content (°Brix) of cherry must was measured using a PAL-BXI ACID F5 digital refractometer (Atago, Tokyo, Japan). Measurements of total sugar, reducing sugar, pH levels, total acidity, total volatile acidity, and alcohol content were determined according to GB/T 15038-2006 [48].

The total amount of phenolic substances was tested with a color-based method called the Folin–Ciocalteu method [49]. The results were expressed in terms of equivalent amounts per liter of the sample. Gallic acid equivalents (mg/L) were used to represent the relevant effect for one type of measurement. For flavonoid substances, a color-testing method with aluminum chloride [50] was employed, and the result was presented as catechin equivalents (mg/L). As for anthocyanin, a method based on different pH levels [51] was utilized to determine its total amount, with the result given as mg/L of wine.

### 3.4. Antioxidant Activity

#### 3.4.1. DPPH Radical-Scavenging Activity

The ability of the sample to scavenge DPPH radicals was evaluated using a method modified from Espín et al. [52]. In brief, 0.5 mL of the diluted wine was combined with 3.5 mL of DPPH• reagent (100 µM). The mixture was then stored in a dark place at ambient temperature for a period of 2 h. Subsequently, the absorbance was determined at a wavelength of 517 nm. The outcomes were expressed as mM of a compound equivalent to Trolox (Trolox-like equivalent units) based on a standard calibration curve.

#### 3.4.2. ABTS Radical-Scavenging Assay

The scavenging ability against ABTS radicals was evaluated using a slightly adjusted protocol compared to what Marfil et al. [53]. reported in 2011. In brief, 0.2 mL of the diluted wine sample was mixed with 6 mL of the ABTS•^+^ solution. The resulting blend was allowed to stand at ambient temperature for half an hour. Subsequently, the absorbance was gauged at a wavelength of 734 nm. The findings were conveyed as millimolar concentrations of a compound with equivalent antioxidant capacity to Trolox (Trolox-like equivalent values) based on a standard calibration graph.

#### 3.4.3. Ferric Ion Reducing Antioxidant Power (FRAP) Assay

The antioxidant capacity measured by the ferric ion reduction assay (FRAP test) was determined following a protocol adapted from Szydłowska-Czerniak et al. [54]. A 0.2 mL aliquot of the appropriately diluted wine was combined with 4.0 mL of the FRAP reagent. This reagent was prepared by mixing 2.5 mL of a 10 mmol/L TPTZ solution, 25 mL of a 0.1 mol/L acetate buffer, and 2.5 mL of a 20 mmol/L FeCl_3_ solution. Subsequently, the produced mixture was maintained at 37 °C for a duration of 10 min. Subsequently, the absorbance was measured at a wavelength of 593 nm. The outcomes were presented as mM Trolox equivalent concentrations based on a standard calibration plot.

### 3.5. Detection of Volatile Compounds Using an Electronic Nose

An electronic nose (PEN 3.5) was employed to conduct an analysis on the various kinds and quantities of volatile chemical entities in the wine test samples. This analysis followed a slightly adjusted method from the one put forward by Song et al. [55] in 2020. In summary, 5 mL of the wine specimen was transferred into a 50 mL airtight purge container and maintained at ambient temperature for an hour. Subsequently, the PEN 3.5 electronic nose was employed to identify volatile substances in these circumstances: 5 s sample preparation time, 120 s sensor cleaning time, 10 s zero return time, sample collection time of 80 s, carrier gas of clean air, and flow rates of 0.3 L/min for both carrier and sample gases. Every specimen underwent a triple analysis. The specific sensitive substances corresponding to the 10 sensors are listed in Supplementary Table S2.

### 3.6. Volatile Compound Analysis via HS-SPME-GC-MS

The species and abundances of volatile substances were analyzed by a method adjusted from Ling et al. [56], with slight alterations. In brief, 5 mL of the wine specimen was pipetted into a 15 mL SPME glass container holding 1.0 g of NaCl and 1 μL of 4-methyl-diamyl alcohol (1.02 mg/L) as a reference standard. Volatile compounds were extracted using a 50/30 μm DVB/CAR/PDMS StableFlex SPME fiber (Supelco, Cat. No. 57329-U). Before the extraction process, the SPME fiber was pre-treated at 250 °C for half an hour. The samples underwent equilibration at 45 °C for 30 min while being continuously stirred.

The division took place using an HP-INNOWAX capillary column (60 m × 0.25 mm × 0.25 μm, Agilent Technologies, Santa Clara, CA, USA). The oven’s temperature was adjusted in this manner: beginning at 40 °C, the temperature was gradually raised to 130 °C at a pace of 5 °C/min, and then further escalated to 160 °C at a speed of 3 °C/min (with a 2 min hold), and finally raised to 230 °C at a rate of 6 °C/min. Helium served as the carrier gas, maintaining a consistent volumetric flow rate of 1.0 mL/min. The injector and ion source’s temperatures were maintained at 230 °C and 250 °C, respectively. The process of mass spectrometry was conducted using electron ionization (EI) mode, utilizing an energy level of 70 eV, with a scan range of 30 to 500 m/z.

Qualitative and Quantitative Analysis: Volatile compounds were pinpointed through spectral matching with the NIST 17 library and by contrasting retention indices (RIs) computed from a linear alkane mixture spanning from C7 to C30 under the same GC-MS experimental setup. Semi-quantification was carried out using 4-methyl-diamyl alcohol as the reference substance. Each analysis was executed three times.

### 3.7. Analysis of Key Volatile Substances

This formula was used to determine the odor activity value (OAV) for each volatile substance: OAV = c/t. Here, c stands for the compound’s concentration (mg/L), and t refers to its odor threshold (mg/L). The odor threshold values were sourced from published research.

To evaluate how much each volatile compound affects the overall aroma, the relative odor activity value (ROAV) was used, as mentioned in earlier studies [57,58].

The maximum OAV value among all compounds was designated as ROAVmax and set to 100 for normalization purposes. ROAV values, ranging from 0 to 100, were calculated using the formula ROAV = (OAVi/OAVmax) × 100, where OAVi represents the OAV of a specific compound and OAVmax is the highest OAV value among all compounds.

Compounds with ROAV values ≥1 were identified as key contributors to the aroma profile, whereas those with ROAV values between 0.1 and 1 were deemed less significant to the overall flavor.

### 3.8. Statistical Analysis

Group differences were assessed through one-way ANOVA. Statistical computations were carried out by employing SPSS 19.0 (SPSS Inc., located in Chicago, IL, USA). Blank samples were included in analytical procedures to correct background interference and ensure quantitative accuracy. PCA (principal component analysis) and OPLS-DA (orthogonal partial least squares discriminant analysis) were implemented using the software SIMCA 14.1. Bar charts were generated using Origin Pro 2021, and radar fingerprints from the electronic nose were created with Excel 2019. A heatmap illustrating the aromatic compounds was constructed using Tbtools. Variable importance in projection (VIP) scores were computed to identify the volatile substances that exhibited disparities. Subsequently, the Kruskal–Wallis rank-sum test (with a significance threshold set at *p* < 0.05) was applied to further validate the outcomes. Each of the experimental trials was repeated three times.

## 4. Conclusions

This study compared the physicochemical properties, antioxidant capacity, and volatile compound profiles of eight sweet cherry wines produced from four cultivars with two alcohol levels. At the same alcohol content, wines from red-fleshed sweet cherry cultivars (FC and RL) contained higher contents of total phenolics, flavonoids, and anthocyanins than those from the yellow-fleshed cultivar. Moreover, within the same cultivar, sweet cherry wines with higher alcohol content showed stronger antioxidant activity than those with lower alcohol content.

An electronic nose and HS-SPME-GC-MS were used to analyze the volatile components in the eight sweet cherry wines. A total of 58 volatile compounds were identified. PCA and OPLS-DA results showed that the samples could be clearly distinguished according to cultivar and alcohol level. Based on VIP > 1 and ROAV analysis, ethyl butanoate, isoamyl acetate, hexyl acetate, methyl decanoate, ethyl benzoate, methyl salicylate, citronellol, benzaldehyde, and eugenol were identified as the key aroma compounds in sweet cherry wines. SMT-11 and RL-6 contained more abundant and diverse key volatile compounds. The contents of key aroma compounds and the final alcohol content differed significantly among sweet cherry wines from different cultivars.

The development of sweet cherry wine is partially limited by high acidity and color instability during storage. The FC cultivar showed the lowest acidity and relatively high antioxidant capacity. In addition, the total content of volatile compounds was higher in low-alcohol wines than in high-alcohol wines. These results indicate that the FC cultivar is more suitable for sweet cherry wine production, especially for low-alcohol sweet cherry wine. Future research will evaluate more sweet cherry cultivars and optimize the appropriate alcohol content for each cultivar to improve wine aroma and quality.

## Figures and Tables

**Table 1:** The physicochemical properties of the eight cherry wines investigated.

Physicochemical Indices	FC-6	RL-6	HM-6	SMT-6	FC-11	RL-11	HM-11	SMT-11
pH	3.73 ± 0.03 d	3.72 ± 0.01 d	3.72 ± 0.01 d	3.56 ± 0.02 f	3.85 ± 0.01 a	3.81 ± 0.01 b	3.76 ± 0.01 c	3.60 ± 0.02 e
Total acid/(g/L)	7.11 ± 0.06 f	8.22 ± 0.10 d	8.09 ± 0.05 e	8.48 ± 0.05 a	7.99 ± 0.06 e	8.91 ± 0.05 a	8.53 ± 0.06 a	8.47 ± 0.05 c
Volatile acid/(g/L)	0.42 ± 0.02 abc	0.45 ± 0.03 a	0.43 ± 0.02 ab	0.44 ± 0.02 ab	0.39 ± 0.02 c	0.43 ± 0.02 ab	0.41 ± 0.02 bc	0.45 ± 0.02 a
Reducing sugar/(g/L)	2.69 ± 0.06 a	1.96 ± 0.03 c	1.75 ± 0.22 d	0.98 ± 0.03 e	2.77 ± 0.03 a	1.98 ± 0.03 c	2.48 ± 0.03 b	1.94 ± 0.06 c
Alcohol/(*v*/*v*)	6.54 ± 0.14 b	6.92 ± 0.17 c	6.08 ± 0.05 d	6.21 ± 0.04 d	11.45 ± 0.03 a	11.22 ± 0.17 ab	11.15 ± 0.09 b	11.28 ± 0.20 ab
TPC/(mg GAE/L)	729.50 ± 6.01 d	834.83 ± 7.35 b	706.07 ± 6.44 f	628.23 ± 5.69 g	832.37 ± 2.29 b	900.90 ± 3.41 a	718.70 ± 2.75 e	809.13 ± 1.76 c
TFC/(mg CAE/L)	60.57 ± 0.55 d	73.77 ± 0.57 b	40.00 ± 1.11 h	50.73 ± 0.60 f	72.00 ± 0.60 c	82.20 ± 0.36 a	44.00 ± 0.98 g	52.40 ± 0.78 e
TAC/(mg/L)	13.68 ± 0.97 c	13.33 ± 1.90 c	7.05 ± 0.71 d	5.11 ± 0.47 e	21.25 ± 1.25 a	16.32 ± 1.48 b	14.21 ± 0.20 c	7.28 ± 0.40 d

Note: FC-11, RL-11, HM-11, and SMT-11—fermented to 11% *v*/*v*; FC-6, HD-6, HM-6, and SMT-6—wines with no high-grade sucrose added. The data are presented as mean ± standard deviation (SD) of three replications. Different letters (a–g) in the same row indicate significant differences (*p* < 0.05) based on the Duncan test.

**Table 2:** The correlation between TPC, TFC, TAC, and antioxidant capacity of cherry wines.

Program	DPPH	ABTS	FRAP	TPC	TFC	TAC
DPPH	1	0.945 **	0.886 **	0.736 *	0.609	0.846 **
ABTS	-	1	0.725 *	0.903 **	0.766 *	0.847 **
FRAP	-	-	1	0.437	0.258	0.672
TPC	-	-	-	1	0.807 *	0.637
TFC	-	-	-	-	1	0.637
TAC	-	-	-	-	-	1

Note: * indicates significant correlation at *p* < 0.05; ** indicates significant correlation at *p* < 0.01.

**Table 3:** Flavor-related compounds and their corresponding ROAV values.

No.	Compounds	Odor Threshold (µg/L)	Odor Description	*p*	VIP	Relative Odor Activity Value (ROAV)							
						FC-6	RL-6	HM-6	SMT-6	FC-11	RL-11	HM-11	SMT-11
1	1-Hexanol	8000 ^a^	Herbaceous	0.006	1.219	0.04	0.05	0.05	0.04	0.04	0.05	0.08	0.04
2	(Z)-3-hexenol	400 ^b^	Herbaceous	0.002	1.210	0.02	0.01	0.01	0.00	0.02	0.01	0.01	0.02
3	1-Decanol	400 ^a^	Fruity, floral, special fatty	0.004	1.083	0.09	0.08	0.05	0.14	0.08	0.07	0.13	0.07
4	Benzyl alcohol	200,000 ^a^	Floral, rose, phenolic	0.003	1.033	0.00	0.01	0.00	0.00	0.00	0.00	0.00	0.01
5	2-Phenylethanol	14,000 ^a^	Floral, rose	0.002	1.004	0.05	0.04	0.03	0.03	0.04	0.05	0.03	0.05
6	1-Dodecanol	1000 ^a^	Floral	0.046	1.021	0.00	0.00	0.00	0.00	0.01	0.00	0.01	0.01
7	Propyl acetate	30,000 ^c^	Sweetish, perfumed	0.015	1.151	0.00	0.00	0.00	0.00	0.00	0.00	0.00	0.00
8	Ethyl butanoate	20 ^e^	Fruity, pineapple	0.003	1.111	0.00	0.00	0.17	4.74	0.00	0.00	9.32	12.01
9	Isoamyl acetate	30 ^a^	Fruity, banana	0.008	1.051	63.73	23.65	21.91	33.40	35.45	46.44	31.06	100.00
10	Methyl octanoate	200 ^a^	Fruity, orange	0.005	1.003	0.21	0.33	0.16	0.32	0.35	0.44	0.23	0.32
11	Isopentyl hexanoate	700 ^d^	Fruity	0.009	1.121	5.42	7.91	0.74	7.44	3.53	3.33	5.05	7.36
12	Methyl decanoate	6 ^e^	Fruity, floral	0.003	1.036	5.33	7.78	0.73	7.32	3.47	3.28	4.97	7.24
13	Ethyl decanoate	200 ^a^	Fruity, fatty, pleasant	0.003	1.005	30.81	17.07	21.05	19.95	12.06	10.83	14.68	20.77
14	Ethyl benzoate	53 ^e^	Mint, fruity	0.004	1.062	2.95	4.40	1.69	2.73	3.32	3.36	3.92	4.71
15	Methyl salicylate	20 ^b^	Peppermint	0.003	1.007	1.52	2.76	0.84	1.41	1.38	1.01	0.56	1.77
16	Octanoic acid	500 ^a^	Rancid	0.004	1.020	0.55	0.57	0.19	0.27	0.20	0.18	0.35	0.31
17	Citronellol	100 ^a^	Lemon, rose	0.002	1.058	0.26	1.51	0.16	0.92	0.57	0.98	0.24	1.24
18	2,4-dimethyl-Benzaldehyde	-	-	0.002	1.001	-	-	-	-	-	-	-	-
19	Eugenol	5 ^b^	Clove	0.004	1.044	2.62	6.29	2.24	2.97	2.97	1.51	0.00	3.98
20	2,4-Di-tert-butylphenol	200 ^a^		0.003	1.026	0.03	0.12	0.88	0.00	0.00	0.06	0.00	0.44

Note: “-”, not determined. ^a^ Odor thresholds were taken from Jiang et al. [42]; ^b^ odor thresholds were taken from Liu et al. [43]; ^c^ odor thresholds were taken from Meilgaard [44]; ^d^ odor thresholds were taken from Pino et al. [45]; ^e^ odor thresholds were taken from Liu et al. [46].

## Data Availability

Dataset available on request from the authors.

## Supplementary Materials

The following supporting information can be downloaded at: https://www.mdpi.com/article/10.3390/molecules31091382/s1.
