# Supplementary material for: Influence of Cherry Cultivar and Ethanol Concentration on the Oenological Properties of Fermented Cherry Wines
Source: Molecules. 2026 Apr 22;31(9):1382. doi: 10.3390/molecules31091382 (PMC13165440; doi:10.3390/molecules31091382)
Supplement: Supplementary file 1 [file molecules-31-01382-s001.zip › molecules-4119346-supplementary.pdf]

Influence of Cherry Cultivar and Ethanol Concentration on the Oenological Properties of Sweet Cherry Wines

Cong Wang[1], Miaomiao Li[1], Liang Li[2], Xutao Wang[2], Bo Li[1*], Yang Yu[1*]

Supplementary materials

Table S1. Basic physicochemical parameters of different sweet cherry cultivars.

No.	Parameters	FC	RL	HM	SMT
1	TSS (°Brix)	17.30±0.20b	17.63±0.15a	15.07±0.12d	15.47±0.12c
2	Total acid (g/L)	8.71±0.04c	8.91±0.04b	8.51±0.07d	9.58±0.02a
3	pH	3.13±0.01b	3.03±0.01c	3.56±0.05a	2.93±0.02d
4	Total sugar (g/L)	120.37±0.81b	126.95±2.34a	113.09±1.19c	117.69±0.92b

Note: The data are presented as mean ± standard deviation of three replications (SD). Different alphabets (a-d) in the same row indicate significant differences ($p < 0.05$) based on the Duncan test.

Table S2. Electronic nose response values of the eight sweet cherry wines.

No.	Sensitive, volatile gases	Response value								VIP
		FC-6	RL-6	HM-6	SMT-6	FC-11	RL-11	HM-11	SMT-11	
W1C	Aromatic	0.27±0.02bc	0.26±0.01c	0.37±0.02a	0.30±0.03b	0.25±0.02c	0.20±0.02d	0.26±0.00c	0.22±0.02d	0.963
W5S	Oxynitride	16.79±2.40a	12.62±1.08cd	11.31±1.49d	12.80±0.69cd	13.75±2.20bcd	15.89±1.10ab	13.77±0.53bcd	14.79±0.60abc	1.101
W3C	Ammonia,aromatic	0.47±0.02b	0.47±0.01b	0.54±0.02a	0.49±0.03b	0.47±0.02b	0.42±0.01c	0.46±0.00b	0.43±0.02c	0.977
W6S	Hydrogen	3.43±0.34a	3.26±0.15ab	2.38±0.10d	2.76±0.23c	3.28±0.11ab	3.64±0.29a	2.95±0.14bc	3.33±0.15a	1.047
W5C	Alkenes,aromatic	0.71±0.01bc	0.71±0.01bc	0.75±0.02a	0.72±0.02b	0.71±0.01c	0.67±0.01e	0.69±0.00cd	0.67±0.01de	1.018
W1S	Alkane	102.66±10.47c	105.72±8.10c	48.73±4.39e	72.95±17.96d	112.04±9.90bc	153.96±16.36a	98.89±6.73c	130.94±16.35b	0.958
W1W	Terpenoids, sulfides	100.61±4.72b	102.99±4.59b	78.71±1.85d	89.92±8.69c	103.28±2.17b	119.16±5.73a	97.43±3.79bc	104.80±2.08b	0.999
W2S	Alcohols, aromatic	82.41±10.02c	94.03±9.95b	46.63±3.04d	70.02±15.46c	98.52±17.78b	141.15±15.56a	91.20±6.92bc	122.40±17.03a	0.993
W2W	organic sulfides	36.70±3.81b	34.09±1.80bcd	27.25±1.09e	30.90±2.38d	34.81±1.90bc	40.40±1.56a	32.87±0.91cd	36.01±0.33bc	1.004
W3S	Alkanes	1.93±0.08b	1.86±0.07b	1.52±0.03d	1.69±0.13c	1.92±0.07b	2.21±0.10a	1.89±0.05b	2.09±0.11a	0.953

Note: The data are presented as mean ± standard deviation of three replications (SD). Different alphabets (a-f) in the same row indicate significant differences ($p < 0.05$) based on the Duncan test.

Table S3. Volatile compound composition and contents of eight sweet cherry wines.

No	Compounds	RI	Relative contents/ (mg/L)							
			FC-6	HD-6	HM-6	SMT-6	FC-11	HD-11	HM-11	SMT-11
1	1-Propanol	1048	4.24±0.26a	0.98±0.06d	0.41±0.05d	1.15±0.22cd	3.26±1.74ab	3.92±1.73ab	0.92±0.31d	2.52±0.30bc
2	Isobutyl alcohol	1103	4.83±0.28a	2.31±1.56f	2.83±0.11e	2.76±0.14e	4.22±0.19b	3.91±0.07c	3.03±0.10de	3.16±0.18d
3	2-Pentanol	1116	0.33±0.03b	0.70±0.06a	0.10±0.17c	-	0.64±0.03a	0.78±0.35a	-	0.79±0.03a
4	1-Butanol	1150	0.09±0.00ab	0.02±0.03bc	0.02±0.02c	0.04±0.01bc	0.07±0.02abc	0.12±0.07a	0.03±0.00bc	0.06±0.02abc
5	Isoamyl alcohol	1213	53.09±2.94b	30.92±1.68d	32.86±1.03d	29.97±1.05d	48.48±1.71c	58.38±0.39a	33.62±0.56d	51.59±3.97bc
6	1-Hexanol	1357	2.40±0.15c	2.59±0.15bc	2.63±0.05b	2.14±0.13b	2.08±0.08d	2.61±0.11bc	4.55±0.06a	1.94±0.13d
7	(Z)-3-hexenol	1373	0.04±0.00ab	0.03±0.01bc	0.03±0.01bc	0.03±0.00bc	-	0.05±0.00a	0.02±0.02c	0.04±0.00ab
8	1-Heptanol	1459	0.21±0.09c	0.11±0.00e	0.15±0.01d	0.15±0.01d	0.29±0.09a	0.25±0.02b	0.11±0.00e	0.16±0.03d
9	2,3-Butanediol	1545	0.44±0.03abc	0.40±0.08bc	0.21±0.02c	0.45±0.10abc	0.72±0.18a	0.71±0.25a	0.52±0.21ab	0.60±0.16ab
10	1-Octanol	1562	0.43±0.02bcd	0.41±0.02d	0.45±0.02abc	0.55±0.01a	0.53±0.14ab	0.41±0.07d	0.50±0.02abc	0.32±0.05d
11	1-Decanol	1765	0.23±0.03b	0.21±0.02bc	0.13±0.03d	0.36±0.04a	0.22±0.02bc	0.18±0.03bc	0.34±0.02a	0.18±0.01c
12	Benzyl alcohol	1893	3.71.±0.03f	7.05±0.38b	2.73±0.16g	4.01±0.38ef	4.35±0.19de	4.60±0.26cd	4.94±0.06c	8.88±0.56a
13	2-Phenylethanol	1929	3.65±0.11b	2.96±0.08c	2.36±0.17e	2.27±0.07e	3.10±0.13c	3.76±0.07b	2.62±0.06d	4.29±292.64a
14	1-Dodecanol	1973	-	-	0.02±0.02ab	0.03±0.00a	0.04±0.00a	0.03±0.03a	0.04±0.02a	0.04±0.00a
	Alcohols (mg/L)		73.69±3.57b	48.700±2.57de	44.93±1.20ef	43.91±1.89f	68.02±1.51c	79.71±1.12a	51.24±0.19d	74.58±4.92b
15	Propyl acetate	987	0.06±0.05bc	0.05±0.04c	0.05±0.01c	0.12±0.01bc	0.11±0.05bc	0.13±0.02bc	0.62±0.09a	0.16±0.09b
16	Ethyl butanoate	1045	-	-	0.02±0.04d	0.62±0.08c	-	-	1.22±0.05b	1.58±0.32a
17	Isoamyl acetate	1122	12.60±3.09b	4.68±0.57d	4.33±0.59d	6.60±1.04cd	7.01±0.49cd	9.18±2.74c	6.14±2.20cd	19.77±1.54a
18	Methyl caproate	1193	0.04±0.00bc	0.09±0.01a	0.02±0.03cd	0.10±0.02a	0.01±0.02a	0.10±0.05	0.07±0.01ab	-
19	Ethyl hexanoate	1240	6.74±0.38d	3.97±0.49e	4.06±0.14e	5.99±0.44d	11.57±0.82b	15.28±1.43a	4.17±0.91e	7.92±0.13c
20	Hexyl acetate	1279	1.28±0.35a	0.71±0.10b	0.49±0.07b	0.87±0.21b	0.56±0.02b	0.78±0.22b	0.89±0.31b	1.34±0.16a
21	Propyl hexanoate	1324	-	-	-	0.03±0.05c	0.20±0.03b	0.35±0.02a	0.02±0.04c	-
22	Ethyl oenanthate	1339	0.06±0.03c	0.05±0.01c	-	0.10±0.01b	0.17±0.01a	0.18±0.05a	0.04±0.01c	-
23	Ethyl 2-hexenoate	1353	0.04±0.00b	-	0.02±0.00c	0.02±0.02c	0.04±0.00b	0.08±0.02a	-	0.04±0.00b
24	Methyl octanoate	1396	0.27±0.039cd	0.43±0.05b	0.21±0.01d	0.42±0.03b	0.46±0.04b	0.58±0.06a	0.31±0.06c	0.42±0.01b
25	Ethyl octanoate	1442	36.00±4.11c	31.20±2.780cd	20.96±2.01e	32.13±1.91cd	47.38±2.34b	57.76±2.92a	27.840±2.78d	35.16±2.30c
26	Isopentyl hexanoate	1465	0.10±0.03ab	0.10±0.01ab	0.10±0.01ab	0.03±0.05c	0.14±0.03a	0.02±0.03c	-	0.09±0.00b
27	Propyl octanoate	1526	0.16±0.02cd	0.16±0.01c	0.07±0.01f	0.14±0.01de	0.25±0.01b	0.30±0.02a	0.13±0.02d	0.18±0.01c
28	Ethyl nonanoate	1542	0.27±0.01c	0.68±0.24a	0.40±0.07bc	0.69±0.15a	0.24±0.06c	0.25±0.05c	0.64±0.06ab	0.72±0.25a
29	Isobutyl octanoate	1558	0.10±0.03a	0.05±0.04b	0.04±0.00b	0.07±0.01ab	0.07±0.02b	0.05±0.00b	0.07±0.01ab	0.06±0.02b
30	Methyl decanoate	1602	0.21±0.01b	0.31±0.03a	0.03±0.01d	0.29±0.02a	0.14±0.02c	0.13±0.02c	0.20±0.03b	0.29±0.02a
31	Ethyl decanoate	1646	40.61±2.11a	22.50±1.91c	2.77±0.48	26.29±1.67b	15.90±2.70e	14.28±1.30e	19.34±2.14d	27.38±0.95b
32	3-Methyl-butyl caprylate	1664	0.79±0.05a	0.71±0.03a	0.35±0.05d	0.50±60.21bc	0.45±0.01c	0.53±0.09bc	0.55±0.04bc	0.58±0.02b
33	Ethyl benzoate	1682	1.03±0.09cd	1.54±0.12a	0.59±0.06e	0.95±0.17d	1.16±0.02c	1.18±0.09c	1.37±0.10b	1.65±0.01a
34	Ethyl succinate	1686	0.26±0.04c	0.13±0.01d	0.13±0.02d	0.09±0.01d	1.15±0.02b	1.85±0.09a	0.12±0.02d	0.26±0.05c
35	Ethyl 9-decenoate	1698	2.10±0.41a	0.97±0.06d	0.32±0.06f	1.00±0.13cd	1.65±0.15b	1.62±0.15b	0.63±0.04e	1.16±0.07c
36	Propyl decanoate	1729	0.13±0.01a	0.09±0.02b	-	0.09±0.01b	0.06±0.02cd	0.03±0.03d	0.07±0.01bc	0.09±0.00b
37	Methyl salicylate	1795	0.20±0.02bc	0.36±0.02a	0.11±0.00d	0.19±0.01c	0.18±0.02c	0.13±0.03d	0.07±0.01e	0.23±0.02b

No	Compounds	RI	Relative contents/ (mg/L)							
			FC-6	HD-6	HM-6	SMT-6	FC-11	HD-11	HM-11	SMT-11
38	Ethyl phenylacetate	1801	0.04±0.00b	0.12±0.02a	0.04±0.00b	0.05±0.02b	0.08±0.00a	0.08±0.07a	0.06±0.01b	0.09±0.00a
39	Ethyl laurate	1852	3.64±0.49a	1.67±0.75bc	0.33±0.05d	2.11±0.53b	1.74±1.03bc	1.04±0.32cd	1.03±0.17cd	1.19±0.26bcd
40	Pentadecanoate	1871	0.30±0.01a	0.23±0.04b	0.02±0.02f	0.18±0.02c	0.11±0.05de	0.07±0.03ef	0.15±0.01cd	0.13±0.00cd
41	Ethyl tetradecanoate	2056	0.20±0.04a	0.18±0.09ab	0.08±0.02b	0.13±0.03ab	0.20±0.08a	0.12±0.04ab	0.12±0.02ab	0.17±0.04ab
42	Ethyl palmitate	2261	0.81±0.05a	0.47±0.09bcd	0.37±0.2cde	0.32±0.02de	0.52±0.07bc	0.27±0.02e	0.29±0.05e	0.59±0.10b
43	Ethyl9-hexadecenoate	2291	0.09±0.00a	0.10±0.03a	0.03±0.01b	0.05±0.02b	0.08±0.00a	0.03±0.03b	0.04±0.02b	0.04±0.00b
	Esters (mg/L)		108.13±4.17a	71.56±6.50d	35.97±2.05e	80.15±4.87c	91.690±4.60b	106.39±4.90a	66.22±6.30d	101.29±4.22a
44	Acetic acid	1463	0.30±0.08cd	0.30±0.12cd	0.17±0.05d	0.47±0.10bc	0.44±0.15bcd	0.75±0.33a	0.63±0.12ab	0.29±0.07cd
45	Propanoic acid	1550	0.04±0.00a	0.04±0.02ab	0.01±0.01b	0.03±0.00ab	0.04±0.00a	0.05±0.00a	0.03±0.00b	0.01±0.02b
46	Hexanoic acid	1859	0.76±0.05b	1.00±0.26a	0.46±0.01cd	0.55±0.10c	0.48±0.03cd	0.35±0.10d	0.59±0.12c	0.60±0.01c
47	Octanoic acid	2071	1.82±0.20e	1.89±0.160de	0.63±0.14de	0.90±0.04cd	0.67±0.07bc	0.61±0.16b	1.18±0.25a	1.05±0.07a
48	Decanoic acid	2283	0.30±0.01a	0.31±0.01a	0.04±0.01d	0.22±0.01b	0.17±0.01c	0.18±0.02c	0.23±0.01b	0.23±0.02b
	Acids (mg/L)		3.22±0.22a	3.53±0.53a	1.32±0.21d	2.12±0.25bc	1.80±0.16cd	1.95±0.18c	2.67±0.45b	2.19±0.14bc
49	Linalool	1553	0.26±0.01d	0.56±0.01c	0.27±0.02d	0.48±0.01c	1.80±0.02b	2.37±0.17a	0.30±0.04d	0.29±0.03d
50	Citronellol	1770	0.17±0.01e	1.00±0.03a	0.10±0.02d	0.61±0.02c	0.38±0.01d	0.64±0.05c	0.16±0.01ef	0.82±0.07b
51	α-Terpineol	1706	0.04±0.00c	0.07±0.01c	0.04±0.01c	0.05±0.02c	0.35±0.02b	0.50±0.06a	0.03±0.00c	0.04±0.00c
52	Geraniol	1857	-	0.22±0.19a	-	0.06±0.11bc	-	0.20±0.05b	-	0.10±0.17bc
53	Butyrolactone	1650	0.13±0.01a	-	-	-	0.07±0.02b	0.08±0.03b	-	-
	Terpenoids and lactones (mg/L)		0.60±0.03e	1.85±0.17c	0.41±0.05e	1.20±0.13d	2.60±0.04b	3.80±0.14a	0.49±0.05e	1.25±0.22d
54	Benzaldehyde	1540	0.14±0.11b	0.95±0.06a	0.06±0.01b	0.15±0.01b	0.04±0.00b	0.07±0.03b	0.14±0.00b	1.07±0.23a
55	2,4-dimethyl-Benzaldehyde	1822	-	0.57±0.37c	0.96±0.21b	-	-	-	-	11.48±0.25a
	Aldehydes(mg/L)		0.14±0.11d	1.51±0.36b	1.02±0.21c	0.15±0.01d	0.04±0.00d	0.07±0.03d	0.14±0.05d	2.55±0.03a
56	Eugenol	2192	0.09±0.00cd	0.21±0.01a	0.07±0.01d	0.10±0.01c	0.10±0.02c	0.05±0.00e	-	0.13±0.00b
57	2,4-Di-tert-butylphenol	2322	0.04±0.08c	0.16±0.05c	1.16±0.11a	-	-	0.08±0.02c	-	0.59±0.13b
	Pheonl (mg/L)		0.13±0.08d	0.37±0.06c	1.23±0.11a	0.10±0.01d	0.10±0.02d	0.13±0.02d	-	0.07±0.13b
58	Geranyl ethyl ether	1514	-	0.03±0.03c	-	-	0.18±0.02b	0.35±0.06a	-	-
	Ethers (mg/L)		-	0.03±0.03c	-	-	0.18±0.02b	0.35±0.06a	-	-
	Total (mg/L)		185.92±6.07ab	127.55±7.78d	84.89±2.53e	127.68±6.32d	164.42±2.96c	192.39±4.19a	120.76±5.96d	182.57±0.83b

Note: "-"not detected. The data are presented as mean ± standard deviation of three replications(SD). Different alphabets (a-f) in the same row indicate significant differences ($p < 0.05$) based on the Duncan's test.

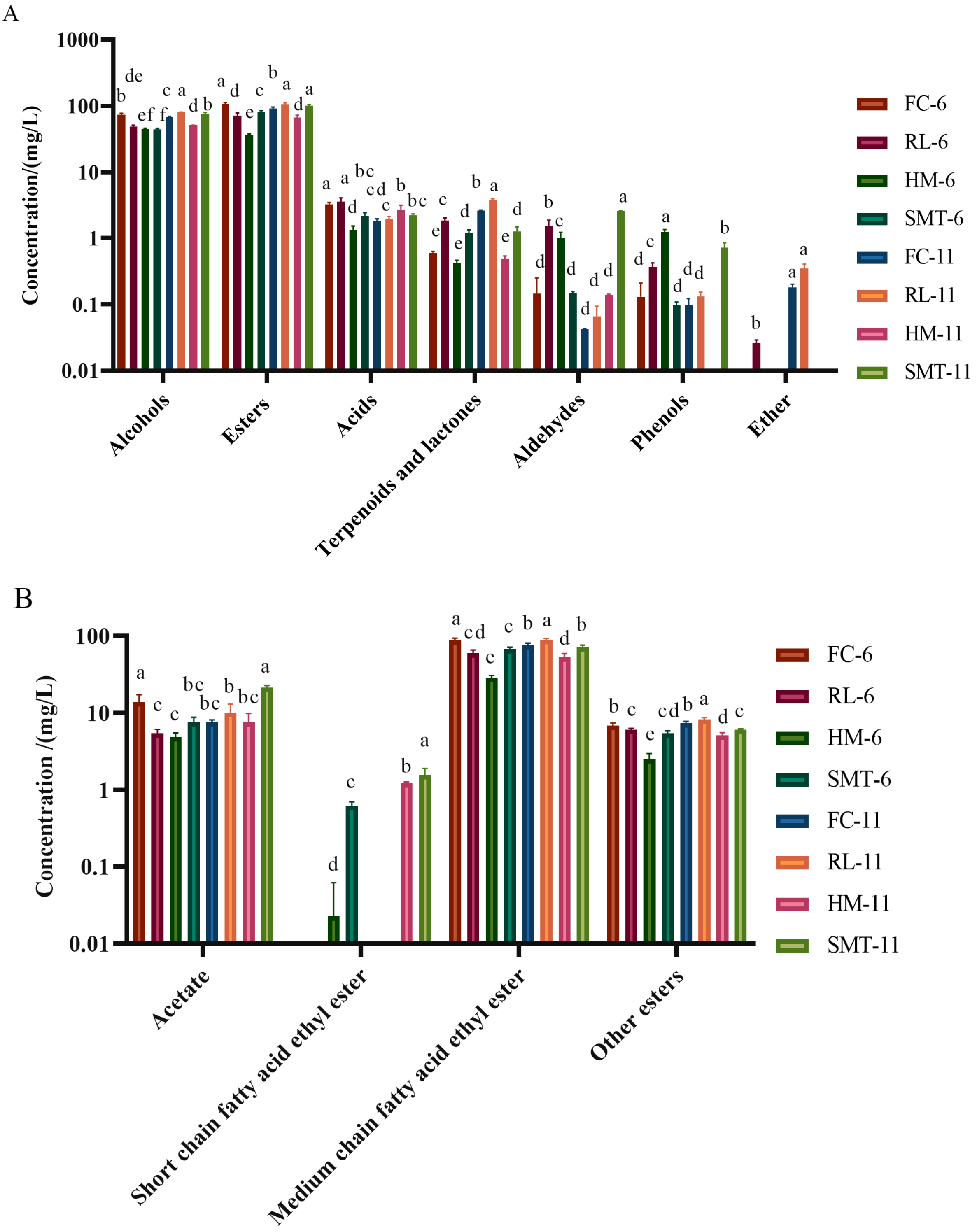

Figure S1. Comparison of total contents of volatile compound classes (A) and ester subclasses (B) in eight sweet cherry wines.Values are expressed as mean ± standard deviation (SD) of three independent technological replicates. Different letters (a-f) indicate significant differences among samples at $p < 0.05$ by Duncan’s multiple range test.
